# The use of low-value imaging: the role of referral practice and access to imaging services in a representative area of Norway

**DOI:** 10.1186/s13244-023-01375-z

**Published:** 2023-02-06

**Authors:** Eivind Richter Andersen, Ingrid Øfsti Brandsæter, Bjørn Morten Hofmann, Elin Kjelle

**Affiliations:** 1 Department of Health Sciences in Gjøvik, The Norwegian University of Science and Technology (NTNU), P.O. Box 1, 2802 Gjøvik, Norway; 2 Centre for Medical Ethics, University of Oslo, Oslo, Norway

**Keywords:** Low-value imaging, Referral practice, Accessibility, Geographical variation

## Abstract

**Background:**

Even though imaging is essential to modern medicine, some examinations are of low value as they do not lead to any change in the management of the patient. The Choosing Wisely (CW) campaign aims to reduce the use of such services. In the Norwegian version of CW, specific magnetic resonance imaging (MRI) of the head, lower back, and knee are amongst others identified as potential low-value examinations. However, referral practice and access to imaging may drive low-value utilisation. By using registry data from 2019 and descriptive analysis, this study aimed to examine the role of referral practice and access to imaging on the use of specific CW-examinations in one representative area in Norway.

**Results:**

A total of 237,554 examinations were performed by four public and two private imaging facilities located within the area. Forty-two percent (19,210/45,289) of all MRI examinations were related to CW. Private imaging centres performed most of the CW-imaging. A total of 3700 referrers were identified, and 2.3% were identified as “high-referrers,” accounting for 33% of all CW-examinations. Referrers’ experience did not influence imaging utilisation. A subset of referrers (“super-referrers,” 0.5%) accounted for 10% of CW-examinations. Distance to service had no impact on the use of CW-examinations.

**Conclusions:**

This study provides valuable insight into the use of imaging and referral practice in one representative area in Norway. A great variation in referral practice was observed. Therefore, targeting referrers with high referral rates may be a promising strategy for reducing the use of low-value imaging.

## Key points

- MRI is frequently used, and a substantial proportion of MRIs are potential of low value.
- A small number of referrers account for a large proportion of potential low-value imaging.
- The use of imaging is not associated with travel distance to imaging facilities or referrers’ experience.
- Targeting “high-referrers” may be a promising strategy for reducing low-value imaging.

## Introduction

Diagnostic imaging is essential to modern medicine as it ultimately contributes to reducing morbidity and mortality [1]. However, some examinations are of low value as they do not lead to any change in diagnosis, treatment, or outcome for the patient [2]. Levin and Rao [3] presented a list of 103 discrete imaging tests considered low-value, and a recent scoping review also identified 87 low-value imaging examinations [4]. The Choosing Wisely (CW) campaign aims to reduce the use of such services, and the campaign has been widely supported in Norway. For example, in the Norwegian version of CW, the following magnetic resonance imaging (MRI) examinations are deemed as low-value and should, in general, not be performed: (1) MRIs for Lower back pain (LBP) without red flags, (2) MRIs for anterior knee pain without mechanical symptoms or effusion, unless there is no improvement in the symptoms after completion of an appropriate rehabilitation programme, and (3) MRIs for uncomplicated headache [5]. These examinations are both resource-intensive and time-consuming. In addition, they can lead to overtreatment and unpleasant experiences for the patient, generate delays for high-value imaging (increasing waiting times), and represent opportunity costs [6]. Hence, it is crucial to document sources and mechanisms (drivers) for low-value utilisation.

In this study, we will focus on two factors that can spur low-value imaging: referral practice and easy access to imaging. Referrals are an important determinant of secondary care utilisation [7, 8], and variation in referral practices may affect the quality of health services, volume, and costs [9]. However, significant variation in referral rates cannot be explained by patient morbidity alone [7], and is partly unexplained [10]. An increase in a “Scan first, ask questions later”-approach, where the decision to perform imaging is based on factors other than patient symptoms and the outcome of clinical examination [11], has been observed. Hence, the decision to refer may be influenced by several factors [10], such as physicians’ knowledge gap, time pressure, physicians’ concern about malpractice (defensive medicine), patient preferences [9], remote consultations, and various innovations designed to speed up diagnostic pathways [11]. Moreover, referral rates are influenced by organisational issues, e.g. whether direct referral to specialist examinations is allowed or not [12]. As wide variations in referral practice may indicate potential low-value referrals, interventions on “high-referrers” is a potential area for quality approvement [7].

Easy access to imaging is another factor that can influence the increased use of low-value imaging. In general, Roemer’s law (“a built bed is a filled bed”) [13] and the more specific “we scan because we can”-doctrine [14] drive supply-sensitive care, including low-value imaging [15]. Hence, variation in access to imaging can influence the use of low-value services, where easy access may increase low-value use [16]. Travelling distance to the imaging facility is one factor influencing access. Norway is a long and narrow country with several remote municipalities where travel time to imaging providers can take several hours [17]. Therefore, variation in service access may result in unwarranted geographical variations, indicating over- or underutilisation. Such variations challenge the basic principle of equal access to health services in Norway [18], the provision of which is a governmental responsibility [19]. Disturbingly, geographical variations in image utilisation are well documented both internationally [20–25], and in Norway [18, 26–28].

The objective of this study was to examine the role of referral practice and access to imaging in terms of travel distance to imaging facilities on the use of CW-imaging. We hypothesised that resource-intensive imaging services (MRIs) are frequently utilised and that a large proportion of examinations conducted are related to examinations included in the Norwegian version of the CW initiative. Furthermore, we hypothesised that individual referral practice and distance to imaging centres would affect CW-examinations’ utilisation. Therefore, by investigating one specific area of Norway as a case, we aimed to examine the following research questions:

- Q1. How were imaging services utilised in general and in relation to the CW-recommendations in 2019?
- Q2. How were the characteristics of referral practice to examinations related to the CW-recommendations in 2019, and what were the characteristics of referrers with high referral rates?
- Q3. How was distance to imaging facilities associated with the use of CW-imaging in 2019?

Data from 2019 was used as this was an ordinary pre-pandemic year.

## Method

### Context

Norway has universal health coverage funded primarily through general taxation [19]. Municipalities are responsible for primary care, and public hospitals and self-employed specialists provide specialist care. Patients pay user fees for most outpatient care, including imaging. However, patients can gain faster access to services through for-profit insurers or out-of-pocket payments through private health providers [19]. In all cases, the patient must be referred to the examination by a doctor, chiropractor, or manual therapist. A radiologist will then assess the appropriateness of a scan and may reject the referral if not indicated.

**Fig. 1:**
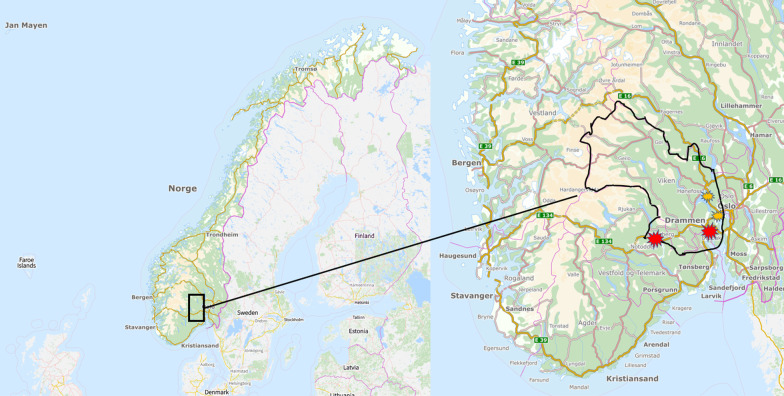
The catchment area of VVHT. The catchment area of VVHT within the black delineation. Red stars indicate locations containing both public and private providers with MRI facilities. Yellow stars indicate the location of public providers with MRI facilities

This study used the catchment area of Vestre Viken hospital trust (VVHT) as a case. This part of Southeast Norway consists of both urban and rural areas with short and long travel distances to doctors and imaging centres. The hospital trust provides specialist health care services in more than 20 municipalities for approximately 500,000 people [29], which is about 10% of the Norwegian population. Two private imaging centres had a contract with regional health authorities in 2019 for delivering radiology services on public terms in this area [17] (see Fig. 1 for details).

### Data collection

Data on all out-patient radiological examinations in 2019 were collected from the Norwegian Health Economics Administration (HELFO) and collected directly from private imaging centres. The requested data included examinations recorded according to the Norwegian Classification of Radiological Procedures (NCRP-codes), patient demographics including age, sex, municipality of residence, and place of examination.

Referrer identification code was collected from the Norwegian Directorate of Health, ensuring that the same referrer working in different offices or departments was identified as the same person. For a subset of referrers identified as “super-referrers,” (see “data preparation and analysis”), the approval year as a health care professional (authorisation), approval year as a medical specialist, age, and gender were collected.

The number of citizens in the municipalities was based on data from Statistics Norway. Travel distance was calculated using Google Maps, estimating the distance in kilometres (km) and driving time in minutes from the patient municipality centre to the nearest imaging centre/hospital with an MRI. When several routes were suggested, the shortest route in km was chosen.

### Data preparation and analysis

Descriptive statistics were used to analyse the data using Microsoft Excel (Version 2207) for Microsoft Office 365 MSO and IBM SPSS Statistics (version 28.0.1.0(142)). As the data from the private imaging centre only contained the name of the examination, e.g. “MRI of the head,” the names were converted to the appropriate NCRP-code. Codes were linked to the anatomical region examined (e.g. MRI of the head), and not to the indication/cause for examinations.

Only data attached to patients living within the catchment area was included. For utilisation of imaging in general, data was grouped by modality, age, and sex.

*“High-referrers”* were defined as referrers with an referral rate to CW-examinations (MRI of head, MRI of lower back or MRI of the knee) at or above the 95th percentile, in accordance with previous literature [8]. *Super-referrers* were defined as referrers at or above the 99th percentile. Both high- and super-referrers were grouped into GPs (doctors specialised in general medicine), Doctors without specialisation, and Specialists. Referrers’ experience was grouped into ‘less experienced’ (less than ten years since approved medical authorisation), and ‘experienced’ (more than ten years’ experience). A Chi-squared-test was performed to examine the differences between these two groups. *p* values less than 0.05 were considered significant.

Distribution of image utilisation in relation to various travel distances was calculated as rates, estimating the distance to the nearest MRI facility by the number of examinations per 1000 inhabitants in the municipalities.

Nuclear medicine (NM) was excluded from the analysis as nuclear imaging is not frequently carried out and was only performed at one hospital in the area.

### Ethics

The study was approved by the Norwegian Centre for Research Data (NSD), approval number 852091.

## Results

### Q1. How were imaging services utilised in general and in relation to the CW-recommendations in 2019?

In total, 258,795 examinations were conducted at the hospitals (*n* = 191,662) or in the private imaging centres (*n* = 67,135) located within the catchment area in 2019. Approximately 14% of the examinations performed at private imaging centres were paid out-of-pocket or through private health insurances.

Four public and two private imaging facilities were located within the catchment area, servicing a population of 494,947 inhabitants. A total of 21,241 examinations were of patients residing outside the catchment area and were excluded from further analysis.

**Fig. 2:**
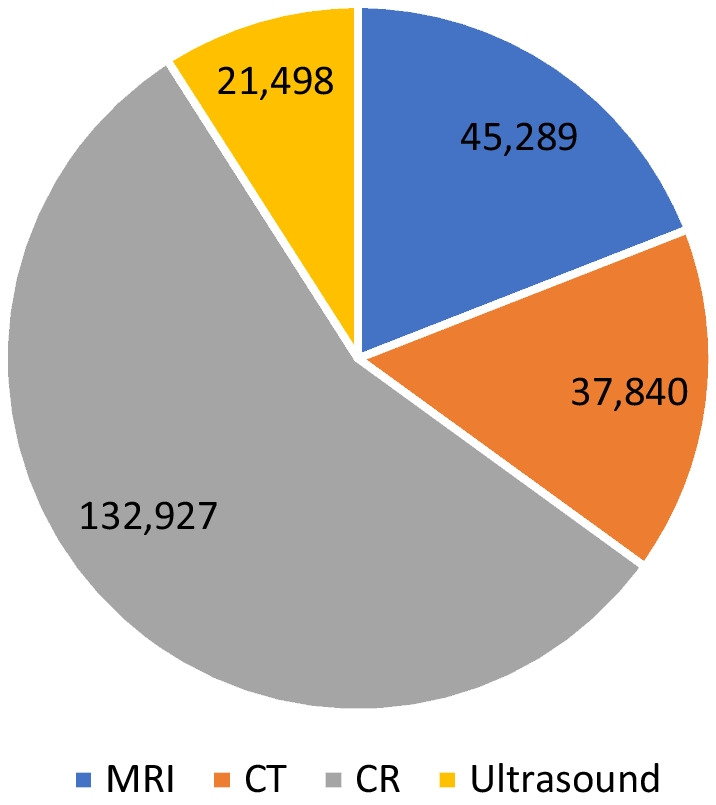
Imaging per modality. Distribution of imaging used per modality

Hence, 237,554 examinations (MRI, computed tomography (CT), Conventional x-ray (CR), and Ultrasound (US)) were conducted on patients living within the catchment area of VVHT in 2019, while 41% (*n* = 97,949) of the examinations were of men and 59% (*n* = 139,605) of women. CR was most frequently used. The overall rate per 1000 inhabitants was as follows: CR = 269, MRI = 92, CT = 76, US = 43. Figure 2 shows the distribution of modalities used in the catchment area.

**Fig. 3:**
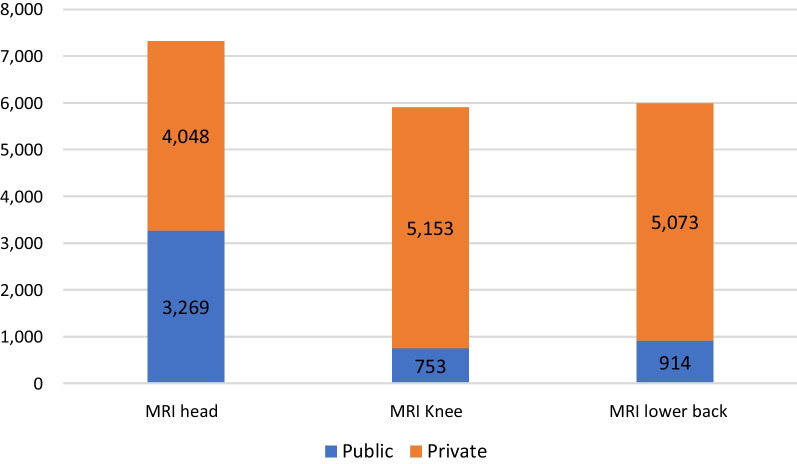
Imaging distribution between public and private providers. Distribution of examinations related to the CW-recommendations between public and private providers

Forty-two percent (19,210/45,289) of all MRI examinations were coded with codes related to the Norwegian CW-recommendations. MRI of the head was the most common of these examinations (*n* = 7317) with 55% performed at private imaging centres. Private imaging centres also accounted for 86% of MRIs of the knee (*n* = 5153) and lower back (*n* = 5073) combined. In total, 74% of the examinations related to the Norwegian CW-recommendations in the catchment area were performed in private imaging centres. Figure 3 presents the distribution of these examinations between public and private providers.

### Q2. How were the characteristics of referral practice to examinations related to the CW-recommendations in 2019, and what were the characteristics of referrers with high referral rates?

A total of 3700 referrers were identified and 1730 of these referred patients to examinations related to the CW-recommendations. Referral rates ranged from 1 to 147 referrals to CW-examinations per referrer in 2019. A total of 86 high-referrers (men *n* = 59, female *n* = 27) were identified, referring ≥ 47 CW-examinations in 2019. This constitutes 2.3% of the total number of referrers. High-referrers were GPs (*n* = 47), doctors without specialisation (*n* = 21), and specialists; neurologists (*n* = 12), orthopaedic surgeons (*n* = 3), ear-nose and throat doctors (*n* = 2), and other (*n* = 1). They accounted for referrals to 33% of all CW-examinations (6270/19,211). High-referrers also referred patients frequently to examinations other than CW-examinations: about 30% of the total number of MRIs, and approximately 15% of the total number of all examinations (all modalities combined). See Table 1 for the specifics.

**Table 1:** Examinations referred by high-referrers

Modality	Examinations referred by high-referrers (*n*)	Examinations performed within the catchment area (*n*)	Percentage of referrals by high-referrers (%)
CT	4420	37,840	12
CR	15,140	132,927	11
US	2449	21,498	11
MRI	13,356	45,289	29
Total	35,746	237,554	15

Number of examinations referred to by high-referrers, the overall number of examinations performed in the catchment area, and the percentage of referrals from high-referrers from the total number of examinations, for each modality

The majority of high-referrers had more than ten years of experience. There was no difference in the referral rates to CW-examinations between the experienced referrer groups and the less experienced referrer groups, *X*^2^ (*df* = 2, *N* = 436) = 3.9, *p* = 0.138. Table 2 shows the number of examinations and the number of referrers for various occupational group, their respective experience, and the rates of the number of examinations per referrer.

**Table 2:** Referral practice by experience

Occupational group	Experience < 10 years			Experience > 10 years		
	CW-examinations (*n*)	Referrers (*n*)	Rate	CW-examinations (*n*)	Referrers (*n*)	Rate
General practitioners	845	11	77	2468	36	69
Doctors without specialisation	747	11	68	610	10	61
Specialists (neurology, orthopaedics, Ear-nose-throat)	69	1	69	1477	16	92
Other	54	1	54	–	–	0
Total	1715	24	268	4555	62	222

Number of examinations and the number of referrers by occupational group and experience as well as examination rates per occupational group

**Fig. 4:**
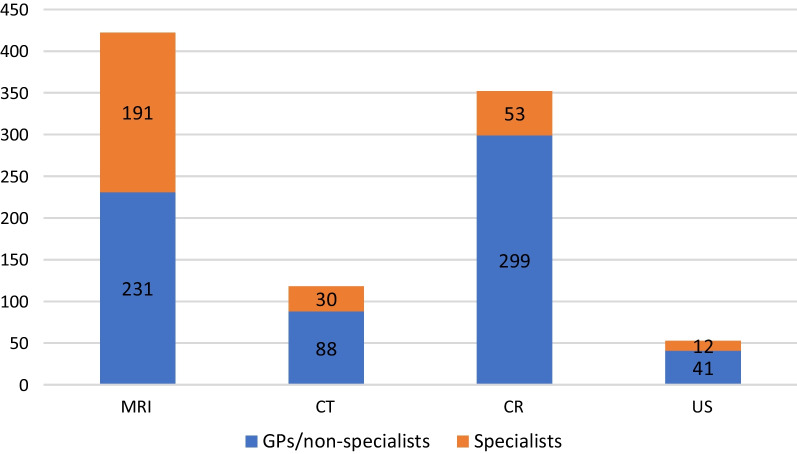
Referral rates per modality for super-referrers. Rates of use (rates = *n* examinations/*n* referrers) among GPs/doctors without specialisation and specialists, deemed as super-referrers per modality

By using the 99th percentile, we identified 17 super-referrers (13 men and 4 women), meaning referrers coupled to more than 87 CW-related-examinations in 2019. This constitutes 0.5% of the total number of referrers. Of these, ten were GPs or doctors without specialisation (59%) while seven were specialists in neurology (*n* = 6) and orthopaedics (*n* = 1). All super-referrers worked in near proximity to an MRI-scanner (within 50 km). Super-referrer GPs/non-specialists referred more to other modalities than neurologists/orthopaedist (differences in rates: CR = 5.6, US = 3.4, CT = 2.9). Referral rates per referrer group (GP/non-specialist and specialists) are provided in Fig. 4. Super-referrers referred to approximately 3.6% of the total amount of imaging performed in the catchment area and accounted for about 10% of the referrals to CW-examinations in 2019.

### *Q3.* How was distance to imaging facilities associated with the use of CW-imaging in 2019?

Access to services related to CW-recommendations varied. Four municipality centres were located more than 100 km away from the nearest MRI-scanner (ranging from 116 to 175 km). Travel time to the nearest MRI facility exceeded one hour for six municipalities (Range 66–163 min). However, most patients resided near MRI facilities (less than 50 km), meaning that estimated driving time was less than one hour. Distance to service had no impact on the use of CW-examinations or MRI utilisation in general, in the catchment area (see Table 3 for specifics).

**Table 3:** Distance to imaging and imaging rates

Travel distance to MRI (km)	Population (*n*)	CW-MRI examinations (*n*)	Rates CW-MRIs per 1000 inhabitants	All MRI examinations (*n*)	Rates all MRIs per 1000 inhabitants
0–49	467,022	17,956	38	42,317	91
50–99	11,724	695	59	1282	109
≥ 100	16,201	559	35	1690	104

Number of inhabitants within various distance ranges to the nearest MRI facility, the number of all MRIs and CW-MRIs, and rates per 1000 inhabitants for CW MRI-examinations and all MRI examinations

## Discussion

The aim of this study was to examine imaging utilisation in general and the use of CW-examinations in particular, and to investigate the role of referral practice, and access to imaging in terms of travel distance to imaging facilities. The setting for the study was the VVHT catchment area. We found that the overall rate per thousand inhabitants varied from 43 for ultrasound to 269 for CR. CR was the most frequently used modality, which is in line with a previous study using data from 2002 [18]. In our data, CT utilisation constitutes 16% of the total imaging used, similar to national data reporting 15% from 2012 to 2015 [27]. Our study documents a 37% increase in MRI utilisation per 1000 inhabitants (rate from 58 to 92) compared to results from 2002 [18], although our study lacks information about in-patient utilisation. Hence, there is reason to believe that the increase in MRI utilisation from 2002 to 2019 is even higher. However, national data from 2016 states that MRI constitutes 22% of the total out-patient imaging in Norway with a rate of 133 per thousand inhabitants [27], while we found MRI use in 19% and a rate of 92 per thousand inhabitants. This may be due to geographical variations nationally [26].

Knowledge about imaging and referral practice is important in identifying areas to target when trying to reduce low-value imaging. Ringberg and colleagues [7] found an overall high referral rate to secondary care and a striking referral range among Norwegian GPs. Moreover, in a study among 480 radiologists, 76% reported that they had received referrals lacking adequate information during the previous work day, and 63% disagreed with the referrer regarding indication [27]. Hence, referral practice may be an important driver for imaging utilisation in general and for low-value imaging in particular. We found great variations in referral practice, and identified 86 of the 3700 (2.3%) referrers as high-referrers. Interestingly, we found no difference in referral rates between less experienced and experienced referrers.

High-referrers referred patients to approximately 33% of all CW-examinations and 15% of all examinations performed in the catchment area, suggesting that our findings are in line with Hong et al. [8] who found that prior imaging patterns and access to equipment were strong predictors for low-value imaging. They also found that the majority of these referrals came from chiropractors and specialists [8]. Interestingly, none of our high-referrers were chiropractors. Therefore, our study confirms earlier findings indicating that Norwegian chiropractors use imaging more sparsely and adhere to guidelines [30]. However, 20% of high-referrers were specialists (mostly neurologists) who may have high referral rates as an MRI of the head is a standard neurological examination for many indications other than the ones included in CW. It is important to emphasise that we cannot identify the rate of low-value referrals, and it is plausible that many of the specialist referrals were warranted. Nonetheless, variation in referral practice may indicate both over and underuse of imaging.

Furthermore, 0.5% of the referrers were super-referrers who referred patients to 3.6% of the examinations in the catchment area. A total of 59% of the super-referrers were GPs or doctors without specialisation, who have an important role as gatekeepers in the healthcare system. Most super-referrers had more than ten years of experience. Kool and colleagues found that 67% of their responding GPs acknowledged that low-value care practice was regularly provided in general practice [31]. There may be many reasons for high referral rates, such as time pressure and patient-related factors [31, 32], (un)familiarity with guidelines [33], and difficulties in handling professional uncertainties [10]. Several strategies are used to avoid imaging overuse [32]. Interventions targeting referrers, including decision support tools, such as the ESR iGuide, guideline implementation, education, feedback to referrers, using various hand-outs, either alone or in combination, have been tried out. Even though the effect of such examinations varies due to contextual and cultural factors, multi-component interventions that include education seem to be more effective than single- component interventions [34]. Our findings of variation in referral practice may indicate flaws in the GPs' role as gatekeepers. However, some GPs might work part-time at emergency clinics, justifying higher imaging rates. On the other hand, the CW-examinations used to identify super-referrers in this study are not typical trauma examinations, making it difficult to explain the high MRI-rates.

Access to services is a driver for utilisation, where the distance to the provider has proven to play a substantial role in healthcare consumption [17]. Also, geographical variations may mean less equal access to radiological services and poor allocation of health resources [18]. MRI and CT utilisation are reported to be slightly higher in urban than rural municipalities [27]. Interestingly, we found no differences in utilisation in relation to travel distance, but a slightly higher MRI utilisation per inhabitant was observed in the groups with longer travel distances. This may indicate that healthcare services fulfil the goal of providing equal healthcare to all citizens despite varying travel distances. Moreover, our findings demonstrate that people in rural areas are willing to travel for services, which is in line with other studies [17]. Higher examination rates for patients in rural parts of the catchment area may be due to a more elderly population [35], thus needing more health services.

This study has a number of strengths. It is the first study addressing referral practice linked to CW-examinations in a Norwegian context. The data used was derived from both public and private imaging providers, which gives a detailed overview of outpatient radiology utilisation in 2019. Moreover, the identification of high- and super-referrers provides a valuable approach to addressing low-value care utilisation. Information about patient municipalities of residence and their use of radiological services also gives valuable insight into the relationship between healthcare utilisation and accessibility.

One limitation of this study is the lack of data for in-patient examinations and a full dataset on examinations fully paid out of pocket or through health insurance. Furthermore, we lack data on referrers owing their own radiological equipment. For instance, one in five chiropractor clinics possess radiological equipment and one in ten have access to ultrasound [30]. The inclusion of this data would give a more complete picture of the imaging utilisation. As this study is based on registry data, we do not have any information on the clinical question or the percentage of findings with impact on patients’ diagnosis, management, or prognosis. Also, the number of referrals is based on the number of examinations connected to the individual referrer and may not reflect the actual number of referrals as several examination codes may have been registered on one referral. Due to the lack of patient identification, some codes may have been used for the same patient at the same examination session, therefore overestimating the number of referrals. However, the great variation in referral practice suggests that referrers with high referral rates may be a warranted target in reducing low-value imaging. Our findings may not be directly transferable to other settings as the study focussed on one specific area. However, the catchment area covers a substantial proportion (10%) of the Norwegian population and is deemed representative of a Norwegian setting as it consists of both urban and rural settlement areas with a variety of distances to health care services.

In this study, our main analysis focussed on high-volume MRIs as reducing such resource-intensive examinations provides a high potential to free resources for high-value examinations. Further research should include examinations using ionizing radiation due to their implications for radio protection.

## Conclusion

This study provides valuable insight into the use of imaging and referral practice in the VVHT catchment area in Norway. In 2019, 237,554 examinations were conducted, and CR was the most frequently used modality. Forty-two percent of all MRI examinations were coded as MRI of the head, knee, or lower back, which are targeted in the Norwegian version of the CW campaign. A total of 2% of all referrers were identified as high-referrers and referred to nearly a third of all CW-MRI examinations. Further, a sub-group of referrers (0.5%) was identified as super-referrers, referring to about 10% of all CW-examinations. Imaging utilisation was not associated with referrers’ experience and distance to services. Targeting referrers with high referral rates may be a promising strategy for reducing the use of low-value imaging.

## Data Availability

The datasets used and/or analysed during the current study are available from the corresponding author on reasonable request.

## Abbreviations

CR: Conventional x-ray
CT: Computed tomography
CW: Choosing Wisely
GP: General practitioner
LBP: Lower back pain
MRI: Magnetic resonance imaging
NCRP-codes: Norwegian Classification of Radiological Procedures
NM: Nuclear medicine
US: Ultrasound
VVHT: Vestre Viken Hospital Trust
